# Eosinophils restrain humoral alloimmunity after lung transplantation

**DOI:** 10.1172/jci.insight.168911

**Published:** 2024-02-08

**Authors:** Zhongcheng Mei, May A. Khalil, Yizhan Guo, Dongge Li, Anirban Banerjee, Yuriko Terada, Yuhei Yokoyama, Christina Kratzmeier, Kelly Chen, Lushen Li, Christine L. Lau, Jean-Paul Courneya, Irina G. Luzina, Sergei P. Atamas, Andrew E. Gelman, Daniel Kreisel, Elizabeth A. Jacobsen, Alexander S. Krupnick

**Affiliations:** 1 Department of Surgery, University of Maryland School of Medicine, Baltimore, Maryland, USA.; 2 Department of Surgery, Washington University in St. Louis, St. Louis, Missouri, USA.; 3 Department of Medicine, University of Maryland School of Medicine, Baltimore, Maryland, USA.; 4 Department of Pathology and Immunology, Washington University in St. Louis, St. Louis, Missouri, USA.; 5 Division of Allergy, Asthma and Clinical Immunology, Mayo Clinic, Scottsdale, Arizona, USA.; 6 Department of Microbiology and Immunology, University of Maryland School of Medicine, Baltimore, Maryland, USA.

**Keywords:** Immunology, Transplantation

## Abstract

While the function of many leukocytes in transplant biology has been well defined, the role of eosinophils is controversial and remains poorly explored. Conflicting data exist regarding eosinophils’ role in alloimmunity. Due to their prevalence in the lung, and their defined role in other pulmonary pathologies such as asthma, we set out to explore the role of eosinophils in the long-term maintenance of the lung allograft. We noted that depletion of eosinophils results in the generation of donor-specific antibodies. Eosinophil depletion increased memory B cell, plasma cell, and antibody-secreting cell differentiation and resulted in de novo generation of follicular germinal centers. Germinal center formation depended on the expansion of CD4^+^Foxp3^–^Bcl6^+^CXCR5^+^PD-1^+^ T follicular helper (Tfh) cells, which increase in number after eosinophil depletion. Mechanistically, we demonstrate that eosinophils prevent Tfh cell generation by acting as the dominant source of IFN-γ in an established lung allograft, thus facilitating Th1 rather than Tfh polarization of naive CD4^+^ T cells. Our data thus describe what we believe is a unique and previously unknown role for eosinophils in maintaining allograft tolerance and suggest that indiscriminate administration of eosinophil-lytic corticosteroids for treatment of acute cellular rejection may inadvertently promote humoral alloimmunity.

## Abstract 2

Eosinophils restrain unwarranted humoral alloimmunity by facilitating Th1, rather than Tfh, differentiation of CD4^+^ T cells through production of IFN-γ in the lung.

## Introduction

Despite the ever-growing acceptance of lung transplantation as therapy for end-stage pulmonary failure, the long-term survival of lungs lags significantly behind that of other organs. This is reflected in higher rates of rejection of the lung compared with heart, kidney, or liver allografts (1). While the diagnosis and treatment of cellular rejection has improved substantially over the last several decades (2), antibody-mediated rejection (AMR) is being increasingly recognized as a substantial cause of morbidity, graft failure, and chronic rejection that is not well controlled by standard immunosuppression targeting cellular immunity (3, 4). The pathogenesis of AMR depends on the activation of allospecific B cells that differentiate toward antibody-secreting cells that generate donor-specific antibodies. Such antibodies bind alloantigen on graft-resident stromal cells and initiate tissue damage through both direct and indirect mechanisms (5–7). Unlike the case for cellular rejection, which has been recognized and studied since the nascent days of organ transplantation, AMR has only recently come to light as a unique and challenging clinical entity (8). Thus, while the etiology, pathogenesis, and therapeutic options for this form of rejection are poorly understood, the clinical need justifies focused research into this process.

Eosinophils are proinflammatory granulocytes that have evolved to combat certain infections and parasites. They are especially enriched at mucosal surfaces such as the gastrointestinal tract and lung airways, but can also be found in other tissues such as bone marrow, thymus, uterus, and adipose tissue (9–11). Both human observational studies and small animal experimental data suggest that eosinophils contribute to rejection of multiple organs such as liver, heart, kidney, and skin grafts (12–19). In the lung, however, we have demonstrated that eosinophils play a critical role in establishing, rather than abrogating, lung

**Conflict of interest:** ASK is founder, president, and board member of AskY Therapeutics. DK serves on the Scientific Advisory Board of Sana Biotechnology.

**Reference information:** *JCI Insight*. 2024;9(3):e168911. https://doi.org/10.1172/jci.insight.168911.

allograft tolerance early in the post-engraftment period (20). Specifically, eosinophils disrupt activation of alloreactive CD8$^+$ T cells and their effector differentiation by limiting T cell receptor (TCR) signaling in an inducible nitric oxide synthase–dependent (iNOS-dependent) fashion (21). To this end, eosinophil depletion prior to engraftment of a lung allograft results in acute cellular rejection despite immunosuppression (20). However, it remains unknown whether eosinophils play a role in the long-term immunoregulation of the lung allograft after the initial engraftment period.

Here, we describe that eosinophils maintain long-term graft homeostasis by restraining humoral alloimmunity. Eosinophil depletion, after tolerance is established through perioperative costimulatory blockade (CSB) of the CD40/CD28 pathways, does not result in cellular rejection but rather leads to increased production of alloreactive antibodies. Mechanistically, we demonstrate that eosinophils restrain humoral alloimmunity by preventing germinal center (GC) formation and T follicular helper (Tfh) cell differentiation. Eosinophils accomplish this by producing high levels of IFN-γ in long-term tolerant lung allografts, thereby facilitating Th1 rather than Tfh differentiation of CD4$^+$ T cells. Our data further advance the understanding of unique immunologic mechanisms controlling long-term lung allograft acceptance and suggest an important and previously unrecognized role for eosinophils in the maintenance of lung homeostasis. In addition, our data indicate that indiscriminate treatment of lung graft recipients with eosinophil-lytic corticosteroids, a clinical strategy common in the treatment of acute cellular rejection, may have unwarranted consequences of promoting, rather than limiting, humoral alloimmunity.

## Results

*Quantitative and qualitative changes in lung-graft-resident eosinophils after engraftment*. To gain an understanding of eosinophils in lung allografts, we transplanted BALB/c (H2$^d$) lungs into fully allogeneic, CSB immunosuppression–treated (22) C57BL/6 (H2$^b$) recipients, as previously described (21, 23–25) and performed serial quantitative and qualitative analysis of graft-resident eosinophils by flow cytometry and RNA sequencing after engraftment (Figure 1A). We noted that eosinophils increased in the graft early after transplantation, but returned to pretransplant levels by day 30 (Figure 1A). Interestingly, such an increase in lung eosinophils after engraftment did not depend on either alloimmunity or CSB, as syngeneic C57BL/6→C57BL/6 transplants demonstrated similar changes in early eosinophil infiltration as well (Supplemental Figure 1A; supplemental material available online with this article; https://doi.org/10.1172/jci.insight.168911DS1). Immunohistochemistry performed on either resting or transplanted lungs confirmed flow cytometry data, with higher numbers of eosinophils on day 7 after engraftment (Figure 1B). While some eosinophils localized to the lung interstitium, a large number could be found in bronchus-associated lymphoid tissue (BALT) as well (arrows in Supplemental Figure 1B). Bulk RNA sequencing of isolated lung eosinophils demonstrated differences in their gene expression at various time points after engraftment (Figure 1C), affecting multiple signaling pathways (Supplemental Figure 1, C–F). Taken together, such data indicate that graft-infiltrating eosinophils are dynamically altered after transplantation and raise the possibility that they may play a role in regulating immune tolerance.

*Delayed eosinophil depletion does not lead to cellular rejection of the allograft*. In order to determine whether eosinophils play a role in the maintenance of lung allograft tolerance, we took advantage of the conditional eosinophil depletion mouse developed by our group, where the diphtheria toxin (DT) receptor is expressed under the control of an eosinophil peroxidase–specific promoter (iPHIL mouse) (26). To accomplish this, we engrafted BALB/c lungs into iPHIL mice on a C57BL/6 background (C57BL/6$^{iPHIL}$ mice), utilizing CSB immunosuppression (22). One month after engraftment, when robust local tolerogenic networks are well established (24), we treated such recipients with either DT or saline for a period of 3–4 weeks prior to analysis (Figure 2A). This regimen did not trigger acute cellular rejection, as the majority of eosinophil-deficient or -sufficient mice had an A0 or A1 International Society for Heart and Lung Transplantation (ISHLT) grade of rejection (Figure 2B). Furthermore, eosinophil depletion did not contribute to quantitative differences in CD4$^+$ or CD8$^+$ T cell infiltration (Figure 2C) or CD4$^+$Foxp3$^+$ T cell accumulation in the graft (Supplemental Figure 2) by flow cytometric analysis. As we have previously shown that BALT plays a major role in the maintenance of tolerance (24), we next considered the possibility that eosinophils may influence the stability of such tertiary lymphoid tissue. Nevertheless, even after 30 days of DT treatment, eosinophil-depleted mice retained BALT with expression of peripheral node addressin (PNAd) (Figure 2D). Thus, unlike in the immediate post-engraftment period (21), eosinophil depletion does not trigger T cell–mediated allograft rejection after tolerance has been established.

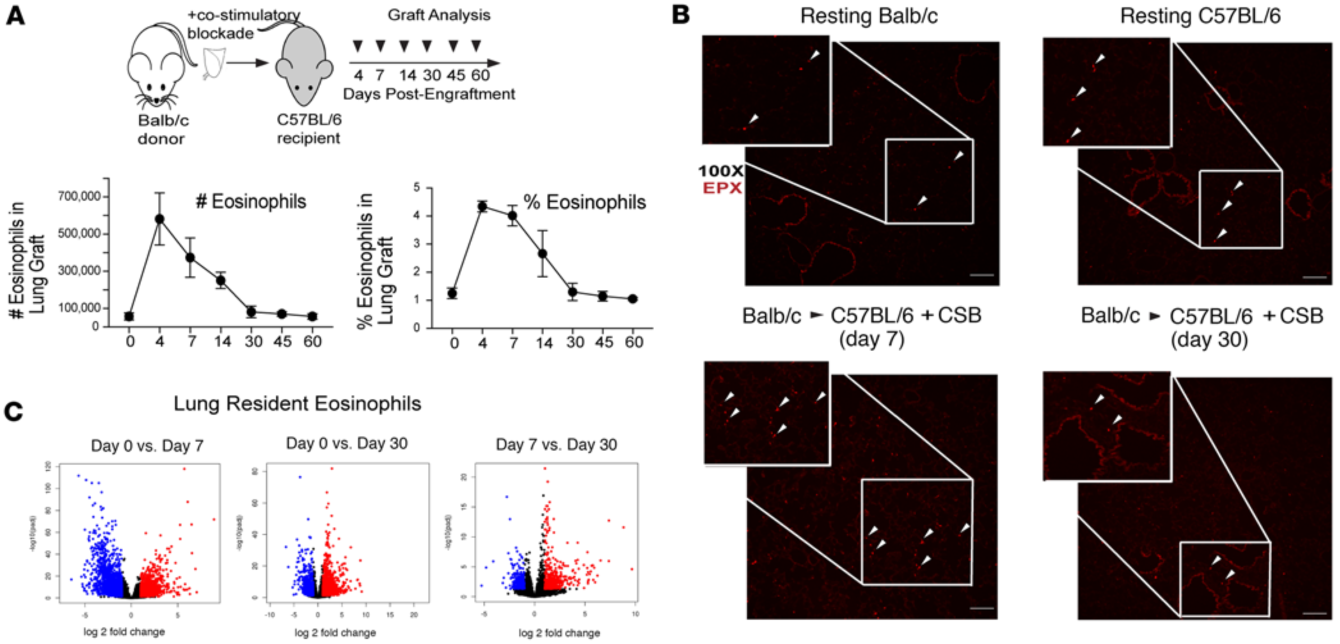

**Figure 1. Lung-graft-resident eosinophils qualitatively change after engraftment.** (**A**) Experimental design (top) and quantification of lung-graft-resident eosinophils expressed as total number or percentage of hematopoietic cells at various time points after transplantation (bottom). (**B**) Immunohistochemical detection of eosinophils using eosinophil-peroxidase staining (red) in resting BALB/c, resting C57BL/6 lungs, and BALB/c→C57BL/6 lung allograft recipients treated with CSB 7 and 30 days after engraftment. Scale bars: 200 μm. (**C**) Volcano plots comparing upregulated (red) and downregulated genes (blue) with a cutoff of absolute $\log_2$(fold change) > 1 and $P < 0.05$ show gene expression in eosinophils isolated at various time points after engraftment. Data in **A** are representative of 3 independent experiments (3–4 mice/group) and are presented as mean ± SEM.

*Eosinophils inhibit B cell differentiation in accepting lung allografts.* To explore the effects of eosinophil depletion further, we next performed single-cell RNA sequencing of DT- or saline-treated BALB/c→C57BL/6$^{iPHIL}$ lung allografts, utilizing the experimental design described in Figure 2A. While some, but limited, changes were evident in multiple clusters of cells, we noted a substantial change in a population corresponding to B cells and antibody-producing cells between eosinophil-sufficient and -deficient mice (Figure 3A and Supplemental Figure 3A). Using flow cytometry, we noted an increase in memory B cells (CD19$^+$Dump$^-$IgD$^-$CD38$^+$GL7$^-$) in DT-treated C57BL/6$^{iPHIL}$ recipient mice (Figure 3B), but limited changes in the number of total B cells or B1 versus B2 differentiation (Supplemental Figure 3B). Significantly higher serum levels of alloreactive antibodies and complement C4d deposition in the lung graft confirmed the activation of humoral immunity upon delayed eosinophil depletion (Figure 3C). No change in antibody levels was evident in resting C57BL/6$^{iPHIL}$ mice that did not undergo transplantation (Supplemental Figure 3C), suggesting a specific role of eosinophils in controlling humoral alloimmunity in the setting of lung transplantation. Taken together, our data demonstrate that eosinophils play a role in the maintenance of established tolerance that differs from that in the immediate postoperative period, where eosinophils prevent cellular rejection (20, 21). Our data also point to a previously unexplored link between eosinophils and humoral alloimmunity after lung transplantation.

Because the transgenic iPHIL mouse colony has been maintained in our laboratories since its original description in 2014 (26), we next decided to validate these findings in wild-type mice from a commercial vendor. As expression of the eotaxin receptor (CCR3) is restricted to eosinophils, the use of an anti-CCR3 depleting antibody has been validated by us as well as others as a reliable and precise method for depletion of this cell population in the absence of off-target side effects (27) (Supplemental Figure 4A). To this end, we transplanted wild-type BALB/c lungs into CSB-treated C57BL/6 recipients. One month later, such tolerant graft recipients were treated with either CCR3-depleting or control antibodies and analyzed for humoral responses 3 weeks later (Figure 4A). Similar to DT-treated C57BL/6$^{iPHIL}$ mice, we noted an increase in (a) serum levels of alloreactive antibodies, (b) C4d deposition in the allografts, and (c) the total number and proportion of memory B cells and GC B cells in the lung grafts in anti-CCR3–treated mice (Figure 4, A and B, and Supplemental Figure 4B). Interestingly, no such changes were evident in the spleen (Supplemental Figure 4C), suggesting that eosinophils affected B cell alloimmune responses locally within the allograft.

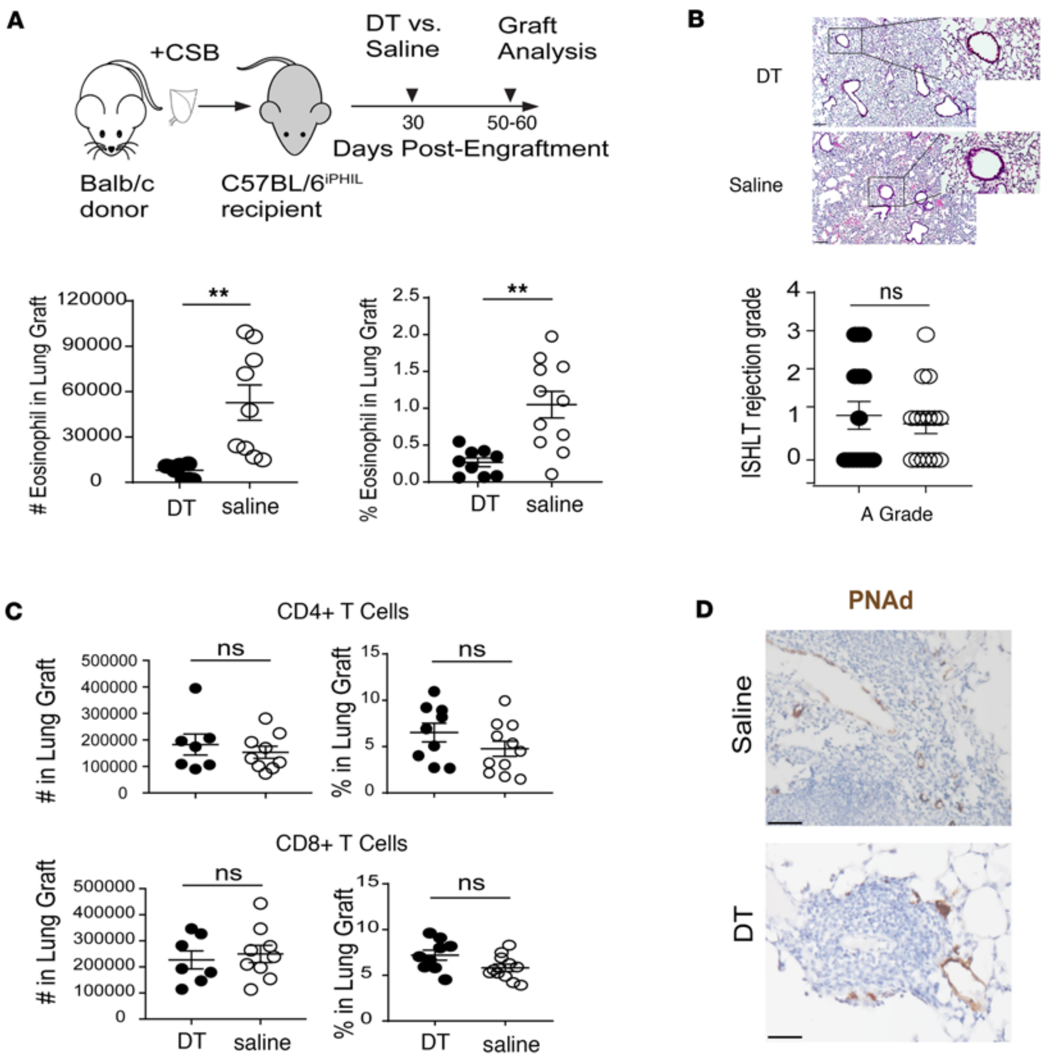

**Figure 2. Delayed eosinophil depletion does not lead to cellular rejection.** (**A**) Experimental design (top) and validation of eosinophil depletion in the BALB/c→C57BL/6$^{iPHIL}$ mouse model (bottom). (**B**) Representative H&E histology and International Society for Heart and Lung Transplantation (ISHLT) rejection grade in the saline- or diphtheria toxin–treated (DT-treated) groups. Scale bars: 200 μm. (**C**) Quantification of CD4$^+$ and CD8$^+$ T cells expressed as total number or percentage of hematopoietic cells after transplantation in the presence or absence of eosinophils. (**D**) Peripheral node addressin (PNAd) immunohistochemistry (brown) in eosinophil-sufficient or -depleted lung allografts. Scale bars: 100 μm. Data are representative of 3 independent experiments (7–11 animals per experiment, with each dot indicating a separate mouse) and are presented as mean ± SEM. NS, $P > 0.05$. $^{**}P < 0.01$ by 2-tailed Student's *t* test (**A**–**C**).

While B cells mature and develop into memory cells locally, long-lived terminal plasma cells (PCs) migrate to the bone marrow after their differentiation from the B cell lineage (28). Bone marrow of eosinophil-depleted mice demonstrated a higher relative abundance of leukocytes expressing the PC-defining marker CD138 (syndecan-1) (29) (Figure 4C). Interestingly, such CD138$^+$ PCs consisted of both B220$^+$ and B220$^{lo/–}$ cell populations, with the CD138$^+$B220$^{lo/–}$ cell population expressing high levels of the PC-critical transcription factor Blimp-1 (30, 31). Taken together, our data demonstrate that in addition to establishing lung allograft tolerance by restraining CD8$^+$ T cell–mediated immunity early after engraftment (21), eosinophils also facilitate long-term tolerance by limiting B cell maturation, thereby restricting allospecific humoral immune responses. Overall, we found these data somewhat surprising, as eosinophils and PCs have been shown to colocalize in the bone marrow and eosinophils have been shown to support B cell and PC differentiation through production of factors such as APRIL, BAFF, and IL-6 in other models (32–34). Thus, the ability of eosinophils to restrain B cell maturation and activation in the context of lung transplantation suggests an unexpected role for this cell population.

*Eosinophils restrain humoral alloimmunity by preventing Tfh cell differentiation*. The development of a productive humoral immune response requires B cell clonal expansion, activation, and affinity maturation. Such processes occur in specialized structures known as GCs (35) where rapidly proliferating B cells and

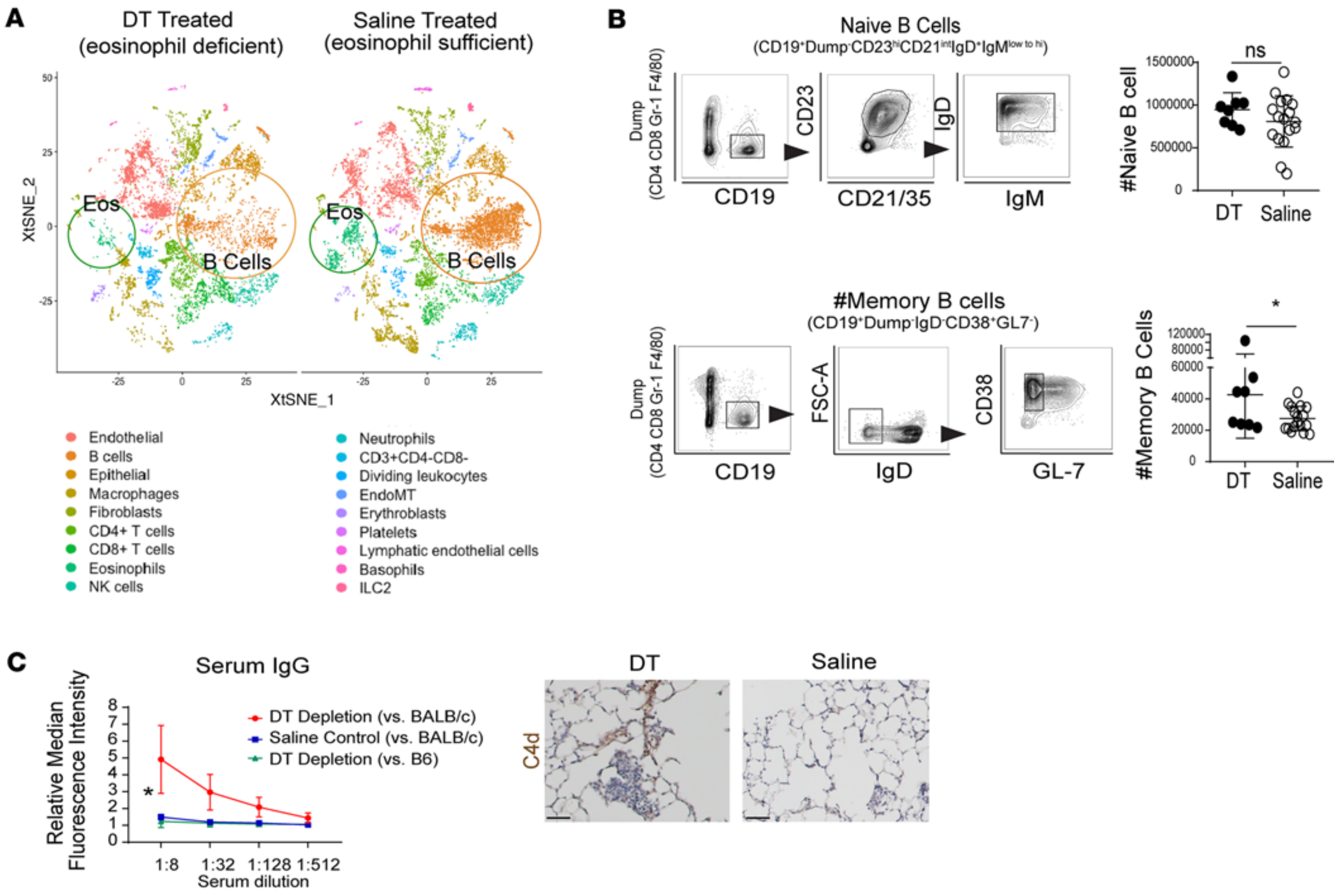

**Figure 3. Eosinophil depletion leads to changes in B cell populations and humor alloimmunity.** (**A**) Single-cell RNA sequencing data of eosinophil-sufficient or -depleted lung grafts as t-SNE plots. EndoMT, endothelial-mesenchymal transition; ILC2, group 2 innate lymphoid cells. (**B**) Quantification of naive $CD19^+Dump^-CD23^{hi}CD21^{int}IgD^+IgM^{lo\ to\ hi}$ and memory ($CD19^+Dump^-IgD^-CD38^+GL7^-$) B cells in lungs of eosinophil-depleted or -sufficient lung grafts. Gating strategy on the left and quantification on the right. (**C**) Alloreactive (red or blue) or autoreactive (green) antibody levels in eosinophil-sufficient or -depleted BALB/c→C57BL/6$^{iPHIL}$–transplanted mice expressed as median fluorescence intensity (MFI) relative to that of standardized serum from a resting, nontransplanted C57BL/6 mouse (top). Immunohistochemistry of lungs from eosinophil-sufficient or -depleted mice stained for complement deposition (C4d staining) (bottom). Brown indicates DAB staining for C4d and blue indicates hematoxylin counterstain of nuclei. Scale bars: 50 μm. Data are representative of 3 independent experiments (9–17 mice/group in **B** and 3–5 mice per group in **C**) and are presented as mean ± SEM. NS, $P > 0.05$. *$P < 0.05$ by 2-tailed Student's *t* test for single-variable differences (**A**–**C**).

supporting cells interact in close proximity. In addition to a defined anatomic structure, consisting of rapidly proliferating GC B cells surrounded by a naive B cell follicular mantle (defined by IgD expression), GCs can be identified by the expression of *N*-glycolylneuraminic acid (the ligand for the antibody GL7) as well as CD21/CD35-expressing B cells and follicular dendritic cells (35, 36). To this end, lungs and mediastinal draining lymph nodes of eosinophil-depleted mice demonstrated increased expression of GL7 and CD21/CD35 (Figure 5A and Supplemental Figure 5A). No differences were evident in the spleen, suggesting that these eosinophil-mediated responses occur locally (Supplemental Figure 5A).

Productive B cell responses and GC formation involve the coordinated interaction of multiple cell types, such as B cells, follicular dendritic cells, and T cells. Based on our previous data demonstrating robust interaction of eosinophils and lung-allograft-infiltrating T cells (21), we next eliminated $CD8^+$ T cells at the time of eosinophil depletion. We noted that alloantibody development still occurred under such conditions (Supplemental Figure 5B). However, we did not observe B cell maturation or alloantibody generation in the absence of $CD4^+$ T cells, even when eosinophils were depleted (Figure 5B and Supplemental Figure 5C). Taken together, such data raise the possibility that eosinophils may regulate B cell responses by impacting the function of $CD4^+$ T cells.

We initially considered the possibility that eosinophils may alter alloreactivity of $CD4^+$ T cells, but were unable to demonstrate any such change in secondary recall response ELISPOT assays (Supplemental Figure 5D). Consistent with the robust tolerance induced by CSB immunosuppression, $CD4^+$ T cells

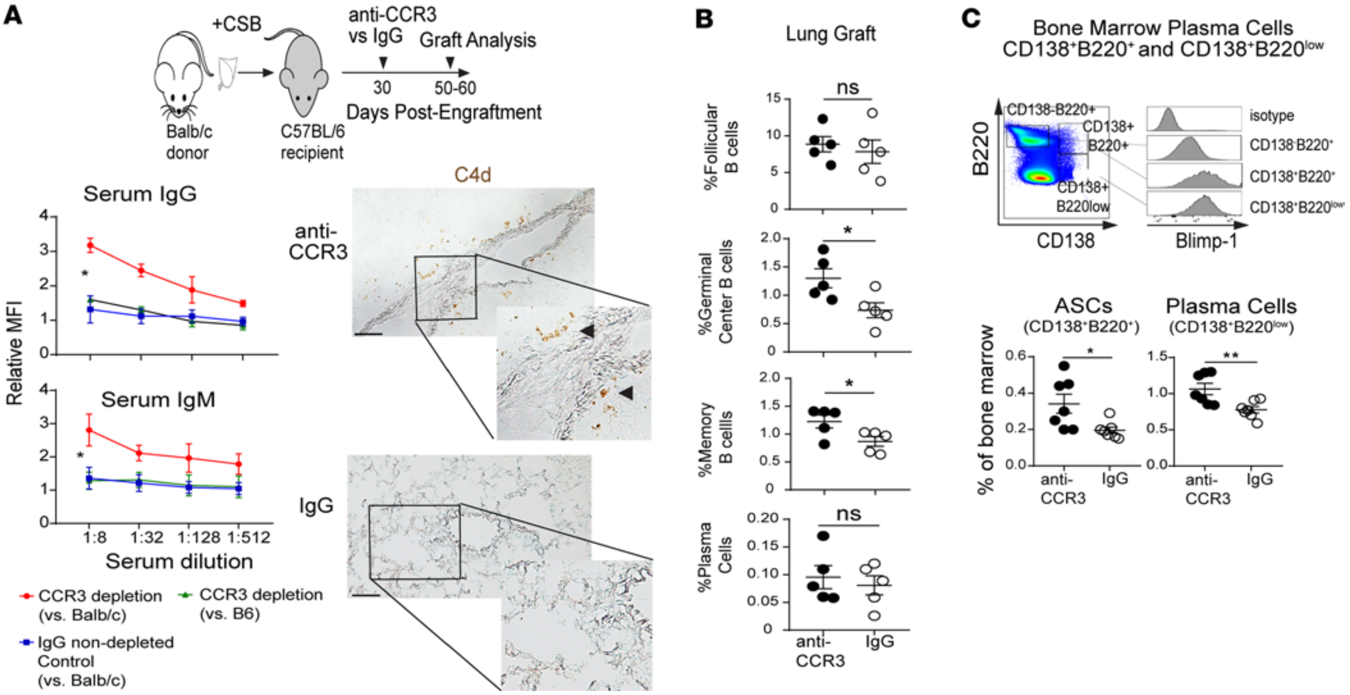

**Figure 4. Eosinophils inhibit B cell differentiation in accepting lung allografts.** (**A**) Experimental design of eosinophil depletion in wild-type mice utilizing anti-CCR3 antibody depletion (top). Relative MFI of serum IgG and IgM in CCR3-depleted or control mice. Allospecific antibodies are depicted as red or blue and autoreactive antibody levels are depicted as green (bottom left). Antibody levels are expressed as MFI relative to that of standardized serum from a resting, nontransplanted C57BL/6 mouse. Immunohistochemistry of lungs from eosinophil sufficient (IgG-treated) or depleted (anti-CCR3–treated) mice stained for complement deposition defined as C4d staining (bottom right). Brown indicates DAB staining. Scale bars: 100 μm. (**B**) Quantification of follicular ($CD19^+Dump^-IgD^+CD23^{hi}CD21^{lo}$), germinal center ($CD19^+Dump^-IgD^+CD23^{hi}CD21^{lo}$), and memory ($CD19^+Dump^-IgD^-CD38^+GL7^-$) B cells as well as plasma cells ($CD138^+B220^{lo}$) as percentage of all $CD45^+$ hematopoietic cells in lungs of anti-CCR3– or control IgG–treated lung allograft recipients. (**C**) Quantification of bone marrow plasma and antibody-secreting cells (ASCs) and Blimp-1 expression in these cell populations. Data are representative of 3 independent experiments (3–5 mice/group in **A**, 5 mice/group in **B**, and 7 mice/group in **C**) and are presented as mean ± SEM. NS, $P > 0.05$. $^*P < 0.05$; $^{**}P < 0.01$ by 2-tailed Student's *t* test for single-variable differences (**A**–**C**).

demonstrated limited to no reactivity to donor-type-specific alloantigen. Gene expression analysis from single-cell RNA sequencing demonstrated limited differences in $CD4^+$ T cells, except for a trend toward Bcl6 upregulation after eosinophil depletion (Supplemental Figure 5E). $CD4^+Foxp3^-Bcl6^+CXCR5^+PD\text{-}1^+$ Tfh cells are specialized providers of B cell help and are essential for GC formation and differentiation of memory B cells (37). We observed an increase in the total number as well as proportion of Tfh cells in eosinophil-depleted lung allografts, locally draining mediastinal lymph nodes (Figure 5C and Supplemental Figure 5F), but not spleens (Supplemental Figure 5F). To study this further, we adoptively transferred naive $CD62L^+CD44^-CD45.1^+$ congenic $CD4^+$ T cells into CSB-treated BALB/c→C57BL/6 (both on a CD45.2 background) lung recipients in the presence or absence of eosinophil depletion. T cells transferred into both eosinophil-sufficient and -deficient grafts had similar increases in expression levels of the regulatory T cell–specific transcription factor Foxp3 and the Th2-specific transcription factor GATA3 (Figure 5D). However, in the presence of eosinophils, higher increases in expression levels of the Th1-defining transcription factor T-bet were evident in the transferred $CD4^+$ T cells, while in eosinophil-depleted mice the Tfh-defining transcription factor Bcl6 was upregulated to a higher degree (Figure 5D). Again, such changes were evident in the lung and mediastinal draining lymph nodes, but not spleens of graft recipients (Supplemental Figure 5G). Our data thus suggest that eosinophils prevent Tfh generation in the setting of lung transplantation by contributing to a Th1-polarized environment. These findings expand on our previous reports where we demonstrated that Th1 polarization is important for induction of lung allograft tolerance (20, 38).

*Eosinophils prevent Tfh differentiation by producing high levels of IFN-γ in accepting lung allografts*. We, as well as others, have demonstrated that eosinophils may alter the phenotype of other leukocytes through direct cellular interactions (21, 32). Expanding on previously described methods, we evaluated the colocalization of eosinophils with various leukocytes in the lung by immunohistochemistry and noted no predilection for $CD4^+$ cell–specific interaction compared to other leukocytes (Figure 6A). While we have previously demonstrated that

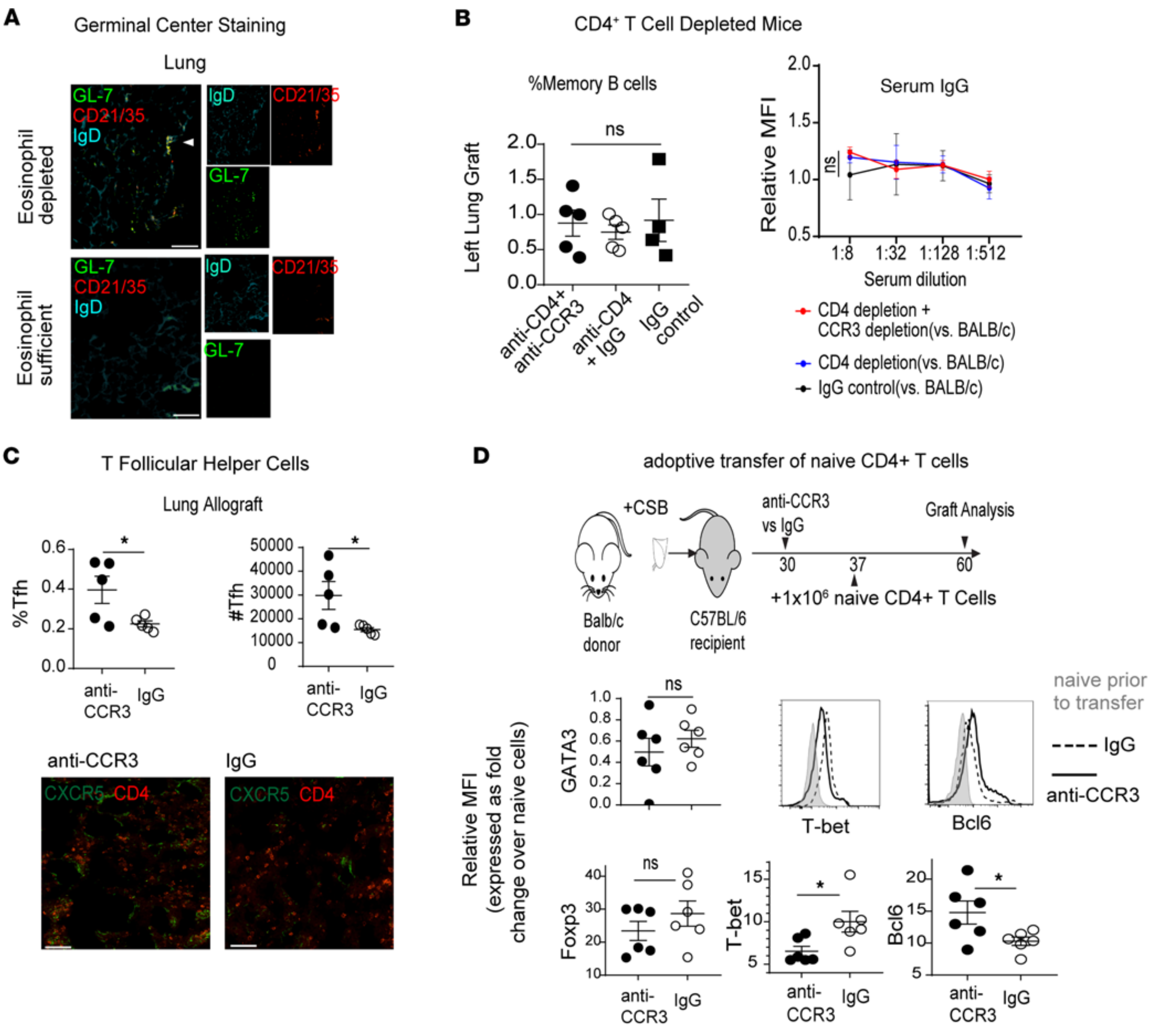

**Figure 5. Eosinophils restrain CD4⁺ Tfh differentiation and germinal center formation.** (**A**) Evaluation of germinal centers as defined by immunohistochemistry for GL7, CD21/CD35, and IgD in lung grafts of eosinophil-sufficient versus -depleted recipients. Representative of 2 separate stainings in 2 sets of mice. Scale bars: 100 μm. (**B**) Quantification of memory (CD19⁺Dump⁻IgD⁻CD38⁺GL7⁻) B cells as percentage of all CD45⁺ hematopoietic cells in lungs of lung allograft recipients depleted of eosinophils and CD4⁺ T cells, only CD4⁺ T cells, or nondepleted controls (left). Allo-antibody levels in BALB/c→C57BL/6-plus-CSB–transplanted mice depleted of both CD4⁺ T cells and eosinophils (red), only CD4⁺ T cells (blue), or treated with IgG control (black) expressed as MFI to relative to that of standardized serum from resting, nontransplanted C57BL/6 mice (right). (**C**) Quantification of Tfh cells (defined as CD45.2⁺CD4⁺Foxp3⁻Bcl6⁺CXCR5⁺PD-1⁺ as either percentage of all CD45⁺ cells or as total number of cells in allograft lungs by flow cytometry (top) and immunohistochemistry (bottom). Scale bars: 50 μm. (**D**) Experimental design of adoptive transfer experiments (top) and relative change in gene expression, compared to starting naive cell population levels, in congenic CD4⁺ T cells transferred to eosinophil-sufficient or -depleted lung allograft recipients (bottom). Data shown as representative histogram plots for T-bet and Bcl6, with gray demonstrating T-bet or Bcl6 levels for naive CD4⁺ T cells prior to transfer; sold line represents levels after transfer into eosinophil depleted mice (treated with anti-CCR3) and dotted line representative of eosinophil-sufficient mice treated with IgG control. Data representative of 3 experiments, 4–6 animals per experiment, with each dot indicating a separate mouse, and are presented as mean ± SEM (**B**–**D**). NS, $P > 0.05$. *$P < 0.05$, **$P < 0.01$ by 1-way ANOVA with Tukey's multiple-comparison test (**B**) or 2-tailed Student's *t* test for single-variable differences (**C** and **D**).

eosinophil expression of iNOS plays a critical role in establishing tolerance (20), there is little evidence linking iNOS to inhibition of Tfh generation. However, data do exist linking proinflammatory cytokine production by cytotoxic lymphocytes to amelioration of Tfh-dependent B cell responses in models of malaria immune evasion (39). To study this in more detail, we expanded on the gene expression analysis of flow cytometrically sorted graft-resident eosinophils described in Figure 1. Parametric gene set enrichment analysis (PGSEA) of Gene Ontology analysis demonstrated that IFN-γ–related responses represented the top pathway active in eosinophils that are present in lung grafts 30 days after transplantation, compared with eosinophils isolated from "naive," resting nontransplanted lungs (Figure 6B). This suggests that an IFN-γ–enriched environment exists in the lung allograft even 30 days after tolerance induction by CSB. Neutralization of IFN-γ in tolerant BALB/c→C57BL/6 lung graft recipients resulted in an increase in the abundance of Tfh cells and memory B cells as well as increases in serum levels of alloantibodies (Figure 6, C and D), suggesting that IFN-γ suppresses Tfh and subsequent humoral alloimmune responses within the lung allograft. Notably, we also observed a temporal increase in IFN-γ expression in eosinophils within tolerant lung grafts after transplantation (Figure 6E). Such data suggest that eosinophils are not just altered by IFN-γ within a tolerant lung allograft, but may serve as a source of this proinflammatory cytokine themselves.

To study this in more detail, we next treated tolerant BALB/c→C57BL/6 lung allograft recipients with in vivo brefeldin, as previously described (40), and analyzed the lungs flow cytometrically for IFN-γ production. To our surprise, the majority of IFN-γ–producing cells in the graft were $CD45^+$Siglec-$F^{hi}$$CD11b^+$ eosinophils (Figure 7A). While NK cells and $CD8^+$ T cells, considered the dominant IFN-γ–producing cells in most immune responses, outnumbered eosinophils in accepting lung grafts (Supplemental Figure 6A), eosinophils expressed higher levels of IFN-γ as well as T-bet than $CD8^+$ T cells, NK cells, or γδ T cells (Figure 7B). Interestingly, eosinophils were not the dominant source of IFN-γ in rejecting lung allografts engrafted without CSB immunosuppression (Supplemental Figure 6B), but remained such in syngeneic transplants (Supplemental Figure 6, C and D). These data suggest that, while at sites of robust immune responses canonical cytotoxic lymphocytes dominate in the production of proinflammatory cytokines, in quiescent lungs eosinophils can serve as the dominant source of IFN-γ.

To further substantiate these data, we measured IFN-γ levels after depletion of either eosinophils (anti-CCR3) or $CD8^+$ T cells and NK cells (combination of anti-CD8 and anti-NK1.1) in tolerant BALB/c→C57BL/6 lungs 30 days after transplantation. Eosinophil depletion reduced IFN-γ levels to those observed in resting untransplanted lungs (Figure 7C). However, the combination of $CD8^+$ T and NK cell depletion did not result in a significant reduction of IFN-γ levels compared to IgG control antibody–treated lung recipients. Consistent with these findings, $CD8^+$ T and NK cell depletion did not alter memory B cell expansion or alloantibody production over IgG-treated control animals (Figure 7, D and E). Adoptive transfer of in vivo–generated $CD4^+$ T cells, enriched for Tfh cells, into CSB-treated BALB/c→C57BL/6 $CD4^{-/-}$ transplants demonstrated no statistically significant differences in memory B cell differentiation or serum alloantibody levels in the presence or absence of eosinophils (Supplemental Figure 7). Collectively, our findings indicate that eosinophils serve as the dominant source of IFN-γ in tolerant lung allografts and restrain humoral alloimmunity by preventing Tfh differentiation and B cell maturation.

## Discussion

In the early era of thoracic organ transplantation, it was postulated that lungs are highly immunogenic, and thus simply require more aggressive immunosuppression to improve long-term survival. Nevertheless, after 35 years of clinical lung transplantation this principle has been put into doubt, as the long-term loss of pulmonary allografts is nearly double that of other solid organs despite more aggressive immunosuppression (41). It is now postulated that the unique immunology of this mucosal barrier organ may require nonconventional immunomodulating strategies rather than global nonspecific downregulation of the immune response (1). This notion is based on our increased understanding of unique antigen presentation, cytokine milieu, and response to stress of the lung compared with other solid organ grafts.

Allorecognition of the lung allograft differs from most other solid organs. Unlike most other solid organs, the lung is rich in antigen-presenting cells and can support local interactions between antigen-presenting cells and T lymphocytes. Early allorecognition of the lung occurs within the graft itself rather than the draining secondary lymphoid organs (24, 42, 43). Immune responses within the graft do not just mediate rejection, but tolerance as well. In the presence of CSB, tolerogenic networks develop within lung, but not heart allografts, that can prevent graft rejection even after retransplantation into a secondary nonimmunosuppressed host (24). Thus, unlike most solid organs, local immune responses play a critical role in lung alloimmunity.

One additional and highly unique aspect of pulmonary allograft biology is the demonstration that production of type 1 proinflammatory cytokines, such as IFN-γ, considered detrimental to other solid organs, plays a critical role in establishing lung allograft tolerance. After murine cardiac transplantation, for example, inhibition of type 1 proinflammatory cytokines prolongs allograft survival (44, 45). Similarly, experimental models of liver allograft transplantation demonstrate that depletion of IFN-γ–producing cells

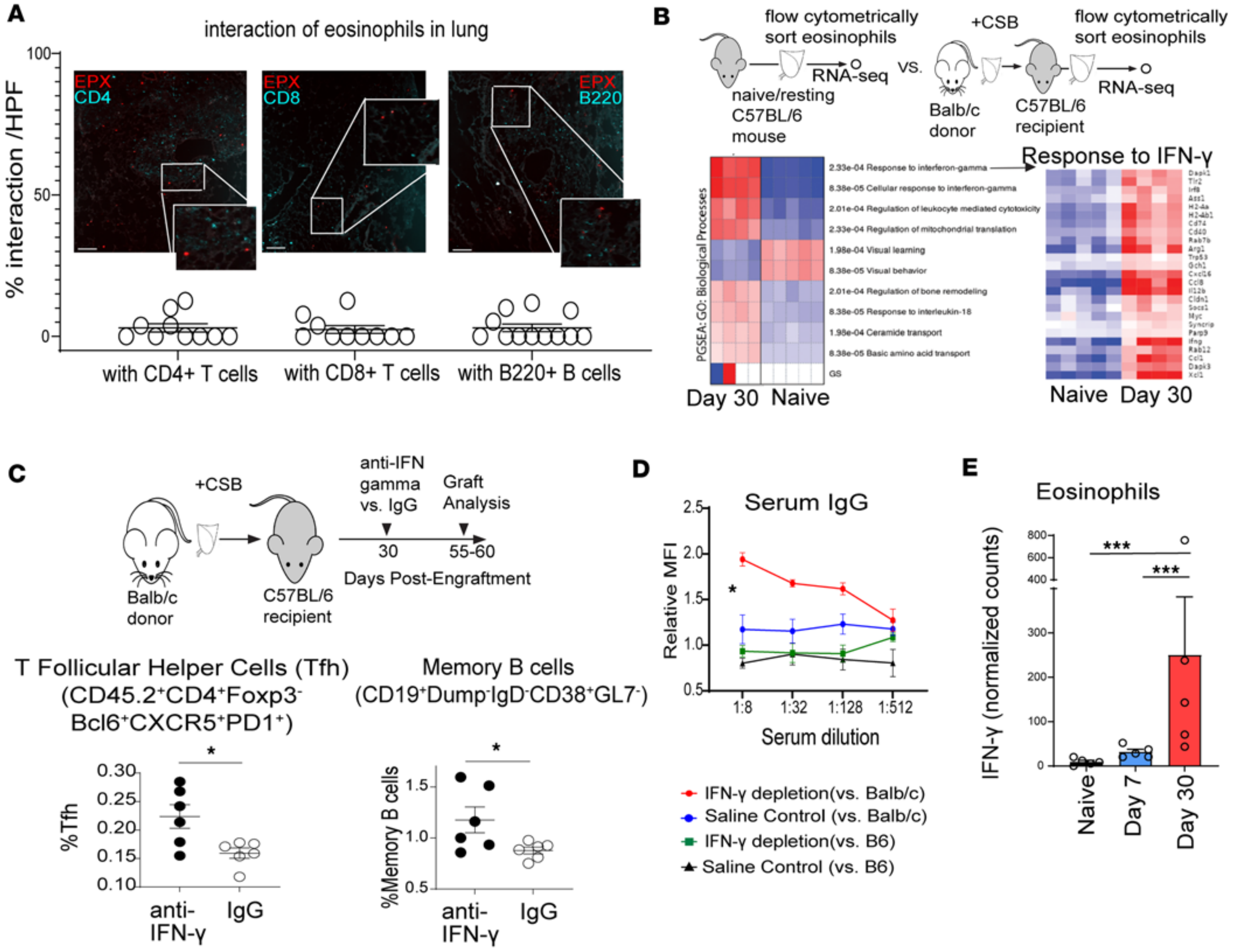

**Figure 6. IFN-γ controls CD4+ Tfh cell numbers and humoral immune responses in the lung allograft.** (**A**) Evaluation of eosinophil (EPX staining, red) and leukocyte (anti-CD4, anti-CD8 or anti-B220, blue) interaction by immunohistochemistry. Data were generated from random sections obtained from 3 separate BALB/c→C57BL/6 transplants treated with CSB 30 days after engraftment. Scale bars: 200 μm. HPF, high-power field. (**B**) Experimental design (top) and parametric gene set enrichment analysis (PGSEA) comparisons in Gene Ontology (GO) pathway gene expression analysis (bottom) of eosinophils isolated from resting lungs or tolerant lung allografts. Representative differential genes for the top GO pathway are shown (bottom right). (**C**) Experimental design (top) and flow cytometric quantification (bottom) of Tfh and memory B cells in the presence of IFN-γ neutralization or IgG control. (**D**) Serum allo- (red and blue) and autoreactive (black and green) IgG levels in the presence of IFN-γ neutralization or IgG control. Data expressed as MFI relative to that of standardized serum from resting, nontransplanted C57BL/6 mice. (**E**) Normalized counts of IFN-γ mRNA by quantitative RNA sequencing in eosinophils isolated from resting lungs versus BALB/c→C57BL/6 lung transplants with CSB. Data are representative of 3 independent experiments; 10 interaction values/group represented in **A**; 3–6 mice/group in **C**–**E**. Data are presented as mean ± SEM. NS, $P > 0.05$; $^*P < 0.05$; $^{***}P < 0.001$ by 1-way ANOVA with Tukey's multiple-comparison test (**A**, **D**, and **E**) or 2-tailed Student's *t* test for single-variable differences (**C**).

ameliorates rejection (46). Lung allografts, on the other hand, rely on such proinflammatory pathways not for rejection, but rather acceptance. Depletion of IFN-γ–producing cells, or antibody-mediated neutralization of this cytokine at the time of engraftment, results in acute cellular rejection rather than prolongation of graft survival. In the lung, IFN-γ initiates a regulatory loop that downregulates T cell activation in the peri-engraftment period (20, 38). In this communication, we now provide evidence that IFN-γ–dependent pathways promote immune quiescence not only early after transplantation by mediating iNOS production and dampening T cell responses, but also at later stages by restraining humoral alloimmunity.

As presented in Figure 7, IFN-γ levels in accepting lung allografts can be 5–10 times higher than those in a resting nontransplanted lung. Our data demonstrate that disruption or depletion of IFN-γ–producing cells after tolerance has been established deregulates humoral immunity, with no substantial effect on cellular rejection. This dependance on type 1 proinflammatory mediators for both establishing and maintaining lung allograft tolerance distinguishes the lung from other allografts. The Strom group, for

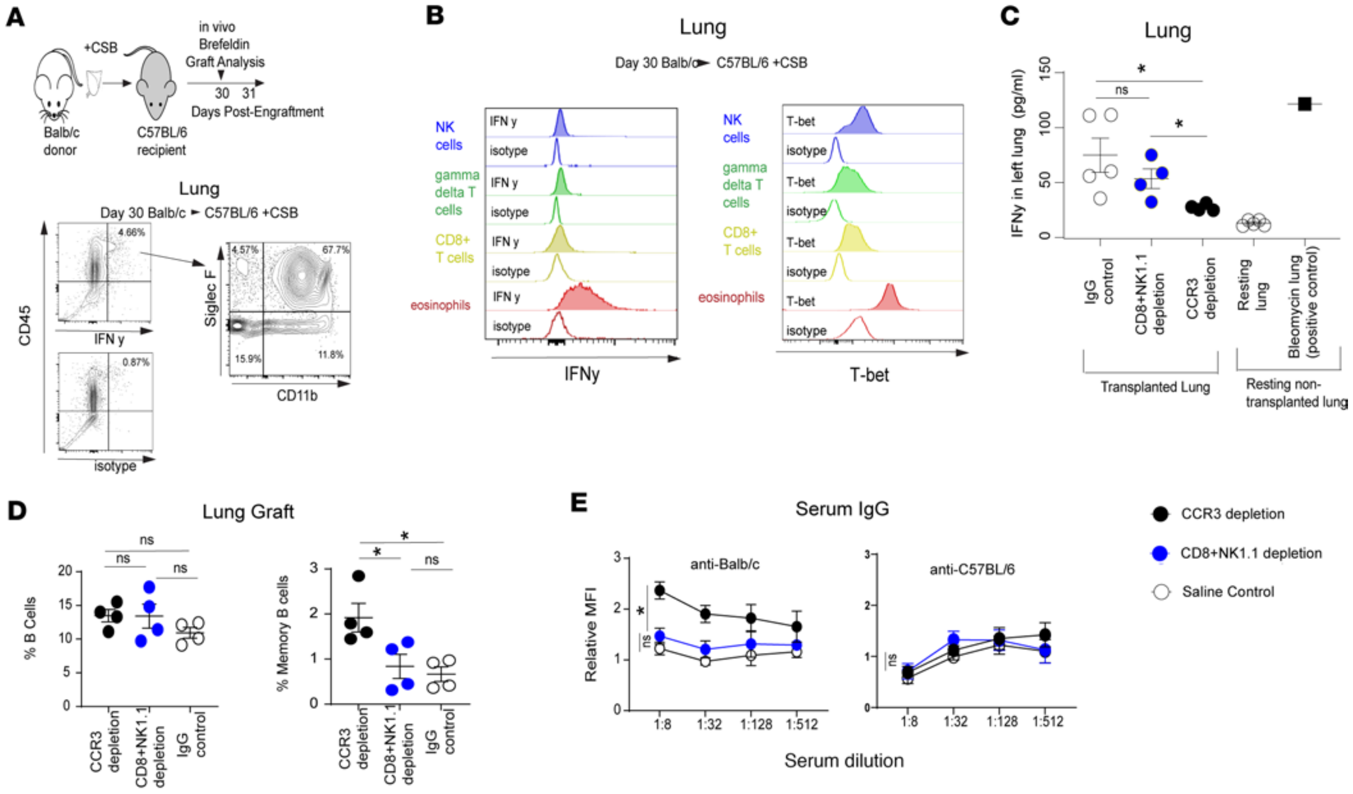

**Figure 7. Eosinophils serve as the main source of IFN-γ in the accepting lung allograft and prevent Tfh differentiation of CD4⁺ T cells.** (**A**) Experimental design (top) and flow cytometric analysis (bottom) of IFN-γ production in lung allografts of tolerant CSB-treated BALB/c→C57BL/6 transplant recipients 30 days after engraftment. (**B**) Relative IFN-γ and T-bet expression in the lung allograft of CSB-treated BALB/c→C57BL/6 recipients 30 days after engraftment (both representative of 2 separate experiments). (**C**) IFN-γ levels in the lungs of BALB/c→C57BL/6 lung transplant recipients treated with IgG versus $CD8^+$ T cell and NK cell depletion versus anti-CCR3 eosinophil depletion. (**D**) Total B cells and memory B cells as well as (**E**) antibody levels in BALB/c→C57BL/6 transplants depleted of either eosinophils or $CD8^+$ T cells/NK cells. Data are representative of 3 independent experiments (4–5 mice/group, except bleomycin lung group in **C** and 3–5 mice/group in **D** and **E**) and are presented as mean ± SEM. NS, $P > 0.05$. *$P < 0.05$ by 1-way ANOVA with Tukey's multiple-comparison test (**C**–**E**).

example, has previously reported that manipulation of the cytokine environment toward type 2 rather than type 1 responses prolongs the survival of islet allografts (47), strengthening the notion that IFN-γ and other proinflammatory cytokines contribute to rejection. A similar process of skewing the type 1 proinflammatory environment toward a type 2 cytokine milieu has been successfully used in experimental models of bone marrow transplant tolerance (48). Along similar lines, adoptive transfer of type 2–polarized cells can facilitate acceptance of cardiac allografts, while transfer of type 1–polarized cells results in rejection (49).

We speculate that such dependence of the lung on type 1 rather than type 2 pathways for tolerance is based on the physiologic necessity of this mucosal barrier organ to discriminate between innocuous environmental exposure and true pathogenic stressors. Unlike other solid organs, the lung has evolved complicated mechanisms for resolution of inflammation that do not exist in hearts, kidneys, and livers (50). Some aspects of such resolution actually depend on proinflammatory mechanisms to initiate the process of healing and repair (51). It is likely that CSB-mediated lung allograft tolerance unwittingly taps into similar pathways to establish robust local tolerogenic networks. Based on experimental data, it is likely that a certain threshold exists where production of proinflammatory cytokines, to a certain point, can limit inflammation. Exuberant inflammatory responses required to clear persistent environmental pathogens, however, do result in bystander tissue damage and lung allograft rejection. This is evident in both experimental models and clinical observational studies demonstrating that *Pseudomonas aeruginosa* infections, which are associated with high production of IFN-γ, can break lung allograft tolerance (52, 53). High levels of IFN-γ present in the rejecting lung allografts similarly support this notion (Supplemental Figure 6). Such immunoregulatory loops may not operate in other transplantable organs, such as hearts, kidneys, or livers, which have not evolved to sense the external milieu and may interpret any increase in proinflammatory cytokines, no matter how small, as indicative of a danger signal.

While allograft rejection has traditionally been considered a cell-dependent process, recent data have demonstrated the importance of B cell activation and antibody production in allograft rejection (8). AMR used to present hyperacutely with graft thrombosis upon implantation due to preformed donor-specific antibodies (54). Humoral alloreactivity can now manifest itself at various time points after transplantation due to de novo generation of allo- and autoreactive (55) antibodies that mediate allograft dysfunction and fibrotic remodeling (56). Multiple immunologic and environment mechanisms, such as loss of $Foxp3^+$ regulatory T cells (25), ischemia-reperfusion injury (57), presensitization due to prior environmental exposure (58), and infection (53), have been linked to AMR. As not all recipients develop AMR, a better understanding of immunologic mechanisms that contribute to this form of rejection may allow for the design of unique strategies to prevent it. Our description that IFN-γ plays a role in restraining humoral alloimmunity in tolerant lung allografts extends our understating of factors that contribute to AMR. Such data also expand our knowledge regarding the unique immunologic aspects of lung transplantation. It is important to point out that our data demonstrate the role of eosinophils in controlling AMR by inhibiting, albeit indirectly, B cell maturation and production of alloantibodies after tolerance has been established. While allospecific antibodies are a hallmark of AMR and are necessary to mediate this form of rejection, other cells execute critical effector functions (59, 60). To this end, reports exist that when NK cells are absent, alloreactive antibodies alone cannot mediate acute rejection of renal allografts (61). The limited tissue damage in eosinophil-deficient grafts contrasts with our recent report where we demonstrated that the depletion of graft-resident $Foxp3^+$ cells triggered the activation of graft-infiltrating B cells, generation of alloreactive antibodies, and histological features of acute lung injury (25). Thus, we speculate that eosinophil depletion, unlike the elimination of $Foxp3^+$ cells, may be insufficient to activate effector cells such as T cells and NK cells (59, 60, 62). Nevertheless, our data uncover a previously unappreciated pathway that can contribute to the generation of alloreactive antibodies, an event that is critical for the development of AMR.

Naive $CD4^+$ T cells demonstrate a remarkable capacity to differentiate based on environmental cues. IFN-γ signaling, traditionally through STAT-1, induces T-bet expression and Th1 differentiation, while in a similar fashion IL-4 signaling through STAT-4 results in GATA-3 upregulation and Th2 differentiation. Other cytokines and environmental cues result in the development of Th17, Th9, or alternative $CD4^+$ T cell subsets (63). The Tfh lineage was originally identified as a CXCR5-expressing $CD4^+$ T cell mainly found in secondary lymphoid organs (64). Tfh cells function nearly exclusively to regulate B cell maturation (65, 66). Unrestrained accumulation of Tfh cells has been linked to antibody-mediated autoimmune diseases (67). We now identify that in the absence of IFN-γ, or IFN-γ–producing eosinophils, naive $CD4^+$ T cells differentiate at a substantially higher rate toward the Tfh lineage in accepting lung allografts (Figure 5, C and D, and Figure 6C), thereby facilitating B cell maturation. In the absence of $CD4^+$ T cells, B cell maturation does not occur, irrespective of whether eosinophils are present or not (Figure 5B). Thus, our data link eosinophil production of IFN-γ to driving $CD4^+$ T cell differentiation away from Tfh toward Th1, which results in inhibition of humoral alloimmunity. While our adoptive transfer studies (Supplemental Figure 7) suggest that eosinophils may not be able to inhibit the function of $CD4^+$ T lymphocytes that have differentiated toward Tfh cells, such an experimental system has several limitations, including a relatively small number of injected cells as well as the possibility for homeostatic expansion with a potential for altered T cell function.

Classic studies have demonstrated that a multistep process is required for Tfh development. Specifically, nascent Tfh cells upregulate Bcl6 following ICOS costimulation delivered by dendritic cells (68). The final commitment and maintenance depend on their interaction with B cells (69). While cytokines associated with Tfh differentiation and function are still being identified, it has been demonstrated that IL-6 and IL-21 enhance, while IL-2, IL-10, and TGF-β inhibit, the generation of this cell population (70–73). However, the role of IFN-γ in the development and function of Tfh cells is poorly described and controversial. Ryg-Cornejo and colleagues, for example, demonstrated that high levels of type 1 proinflammatory cytokines such as IFN-γ associated with malaria infection prevented Tfh differentiation and humoral responses to this parasite. While precursor Tfh-like cells with high levels of Bcl6 were generated during infection, such cells expressed low levels of PD-1 and CXCR5 and failed to facilitate GC responses or B cell maturation (39). Inhibition of IFN-γ and proinflammatory cytokines facilitated B cell maturation and productive antimalaria humoral responses. While some have confirmed these findings in infectious disease models (74) in other models, such as that of lupus-associated autoimmunity, investigators have suggested that IFN-γ signaling can facilitate rather than inhibit Tfh development and function. For example, Lee and colleagues demonstrated that increased IFN-γ signaling resulted in Bcl6 overexpression, Tfh generation, B cell differentiation, and production of pathogenic antibodies. Blockade of IFN-γ in this model reduced Tfh generation and pathology (75). Our data, using a model of lung allograft tolerance, demonstrate that IFN-γ interferes with the generation of Tfh cells and decreases accumulation of $CD4^+Foxp3^-Bcl6^+CXCR5^+PD\text{-}1^+$ cells. Taken together with published data, factors responsible for the generation, maturation, and maintenance of this cell population may be context and site dependent.

It did not escape us that the approximate doubling of bone marrow–resident plasma and antibody-producing cells, evident in eosinophil-depleted mice illustrated in Figure 4C, does not correlate with the 3- to 4-fold increase in alloantibody levels shown in Figure 4A. While we looked for, and did not detect, and increase in plasma cells in the lung allografts or spleens of eosinophil-depleted compared to control mice (Supplemental Figure 4, B and C), we saw substantially more memory B cells, which can also produce alloantibodies (Figure 4B). Our data could also be explained by other possible sites harboring the development and residence of alloantibody-producing cells. This could include mucosal surfaces of the gut (76) or other noncanonical sites (77). Alternatively, the differences between antibody titers, as determined by binding to allogeneic splenocytes, and quantification of antibody-producing cells between eosinophil-depleted and -sufficient mice, could be the result of differences in affinity between antibodies produced by various subtypes of leukocytes involved in humoral immunity (78).

Our data further expand our understanding of eosinophil biology. The formerly accepted notion that eosinophils are a cell population whose only function is to clear parasitic infections and contribute to Th2-mediated disease processes such as asthma has been replaced by the notion that these granulocytes can demonstrate extreme plasticity in phenotype and function based on environmental cues. Specific activation of eosinophils can thus allow them to serve as the dominant source of nitric oxide in the lung allograft (20, 21). They have also been shown to regulate adipose tissue function, enhance neuronal growth, and increasingly are found to have a potential role in cancer cell killing, through unique immune functions as well as mediator release (e.g., lipids or enzymes) (79, 80). In addition, eosinophils can also modulate T cells in several disease models, such as inducing subsets of T regulatory cells in the gastrointestinal tract via TGF-β (81), promoting restoration of T cell production in the thymus after damage (82), and increasing tumor killing through modulation of the T cell activation state (80). Consistent with our findings, eosinophils have also been shown to serve as a source of Th1 cytokines. For example, Legrand and colleagues have demonstrated that eosinophils can kill tumor cells via granzyme and TNF-α (83), a function typically attributed to professional effector cells such as T cells and NK cells. Others have demonstrated that eosinophil production of IFN-γ can mediate asthma-like lung inflammation in the absence of other leukocytes (84). Similar to this work, we now demonstrate that eosinophil-derived IFN-γ is responsible for restraining humoral alloimmunity in a mouse model of lung transplantation.

It remains puzzling why eosinophils, rather than other effector cells such as NK cells or $CD8^+$ T cells, serve as the dominant source of IFN-γ in long-term-tolerant lung allografts. Specifically, in the early period of engraftment $CD8^+$ T cell–derived IFN-γ plays a critical role in tolerance induction (38). It is possible that this dichotomy is related to the physiology of proinflammatory cytokine production between eosinophils and canonical professional effector cells. For example, NK cells, while containing ample mRNA for cytotoxic mediators, maintain strict translational control and do not produce or secrete such proteins without further stimulation (85). Eosinophils, on the other hand, contain ample preformed stores of cytokines, including IFN-γ and cytotoxic mediators, that can be secreted with minimal stimulation (86). This has been demonstrated in both human (87) and mouse eosinophils (88). It is thus possible that unique environmental factors in the tolerant lung allograft inhibit $CD8^+$ T cell– and NK cell–mediated production of cytokines, but eosinophils escape such control. Nevertheless, our data further extend our understanding of the unique immunological networks that regulate lung transplant tolerance and reinforce the idea that indiscriminate immunosuppression may harm the long-term viability of this organ.

## Methods

*Animals*. Male BALB/c, C57BL/6 (B6), B6.SJL/BoyJ CD45.1, and B6.129S2-*Cd4*$^{tm1Mak}$/J mice were purchased from the Jackson Laboratory. C57BL/6$^{iPHIL}$ (eosinophil DT receptor mice) were generated by our group as previously described (26). All mice were kept in the same room of the same vivarium (Program in Comparative Medicine, University of Maryland, Baltimore) after delivery from vendors with the same diet and water supply before being used for each experiment.

Orthotopic left lung transplants were performed using either the BALB/c→C57BL/6 or BALB/c→C-57BL/6$^{iPHIL}$ strain combinations as described throughout the text. For most experiments, except where defined, tolerance was induced with CSB consisting of 250 μg of anti-CD40L antibody (MR1) on postoperative day 0 and 200 μg of mouse recombinant CTLA4-Ig on postoperative 2 (both purchased from BioXCell and given i.p.). For select experiments, recipient mice were treated with depleting antibodies for either elimination of eosinophils (anti-CCR3, clone 6S2-19-4, 200 μg/mouse, given 3 times per week) or IFN-γ neutralization (clone H22), both from BioXCell. Depletion of eosinophils in C57BL/6$^{iPHIL}$ mice was accomplished as previously described (26). Anti-CD4 (clone GK1.5, 200 μg/mouse, once per week), anti-CD8 (clone YTS 169.4, 100 μg/mouse, once per week), and anti-NK1.1 (clone PK136, 100 μg/mouse, once per week) were purchased from BioXCell and given i.p.

*Histology, immunofluorescence, and immunohistochemistry*. For H&E staining, lung grafts were harvested and fixed for 2 days in 10% buffered formalin (Thermo Fisher Scientific) and then transferred to 70% ethanol. Samples were embedded in paraffin and then stained with H&E. A lung pathologist blinded to the experimental condition graded acute rejection according to the ISHLT criteria (89).

For immunofluorescence experiments, mouse lymph nodes were harvested and frozen in OCT (Sakura Finetek) on dry ice. Mouse lungs were intratracheally and interstitially injected with 10% formalin/OCT (1:1) solution before being frozen in OCT. Cryosections (7 μm) were fixed with cold acetone/methanol (1:1) solution for 5 minutes. Antibodies were diluted according to the manufacturer's protocol. After staining with primary antibodies, sections were blocked with 10% serum of the secondary antibody host and incubated with secondary antibodies for 60 minutes. Slides were fixed with 4% paraformaldehyde solution followed by 1% glycerol incubation for 5 minutes. ProLong Gold Antifade Mountant (catalog P36930, Thermo Fisher Scientific) was added before mounting the cover slides. Images were acquired with the EVOS FL Auto 2 and Leica DM6 B Imaging system and analyzed with LAS X analysis software (Leica).

Primary antibodies used were rat anti–mouse ER-TR7 (clone sc-73355, Santa Cruz), FITC anti–mouse/human GL7 (clone GL7, BioLegend), phycoerythrin (PE) rat anti–mouse CD21/CD35 (clone eBio4E3, Thermo Fisher Scientific), purified rat anti–mouse IgD (clone 11-26c.2a, BD Biosciences), purified rat anti–mouse CXCR5 (clone 2G8, BD Biosciences), PE rat anti–mouse CD4 (clone H129.19, BioLegend), biotinylated mouse anti–mouse EPX (1:1000; Mayo Clinic, Scottsdale, Arizona, USA), Alexa Fluor 647 rat anti–mouse CD4 (clone RM4-5, BD Biosciences), Alexa Fluor 647 rat anti–mouse CD8a (clone 53-6.7, BD Biosciences), Alexa Fluor 647 rat anti–mouse B220 (clone RA3-6B2, BD Biosciences), and polyclonal rabbit anti–mouse C4d (catalog HP8033, Hycult Biotech). Secondary antibodies used were allophycocyanin (APC) donkey anti–rat IgG (1:400; Jackson ImmunoResearch), Alexa Fluor 488 donkey anti–rat IgG (1:400; Jackson ImmunoResearch), PE anti-biotin (clone 1D4-C5, BioLegend), Alexa Fluor 488 goat anti-mouse (1:1000; Thermo Fisher Scientific), and Alexa Fluor 555 goat anti-rabbit (1:1000; Cell Signaling Technology).

For immunohistochemistry, formalin-fixed, 5-μm sections of paraffin-embedded specimens were deparaffinized and rehydrated. Following antigen retrieval in citrate buffer (pH 6.0, Dako), endogenous peroxide activity was quenched with 3% $H_2O_2$. Primary antibodies used were anti–eosinophil peroxidase antibody (1:400; clone MM25-82.2, Mayo Clinic, Scottsdale, Arizona, USA) and rat monoclonal anti–mouse PNAd (1:100; clone MECA-79, BD Biosciences). Secondary antibodies used were biotinylated goat anti–mouse (EPX) IgG (BA-9200; 1:400) and biotinylated goat anti–rat (PNAd) IgG (1:500; both from Thermo Fisher Scientific).

*Bulk RNA isolation, sequencing, and analysis*. Eosinophils were isolated by flow cytometric sorting from pooled mice ($n$ = 5) and for 4–5 replicates of each condition: naive, CSB-treated transplant (day 7), and CSB-treated transplant (day 30). After raw data were trimmed and processed, differentially expressed genes were determined using DESeq2 (https://github.com/thelovelab/DESeq2), with an FDR of less than 0.05 and an absolute $\log_2$(fold change) of greater than 1. Functional pathway analysis and PGSEA was completed with online analysis tools such as iDEP.96, Reactome, and ToppGene (see below).

RNA sequencing library preparation used the NEBNext Ultra RNA Library Prep Kit for Illumina by following the manufacturer's recommendations (New England Biolabs). Sequencing libraries were validated using the TapeStation 4200 (Agilent Technologies) and quantified by using Qubit 2.0 fluorometer (Invitrogen) as well as by quantitative PCR (Applied Biosystems).

The sequencing libraries were multiplexed and clustered on 1 lane of a flow cell and loaded on the Illumina HiSeq instrument according to manufacturer's instructions. The samples were sequenced using a 2 × 150 paired-end configuration. Image analysis and base calling were conducted by the HiSeq Control Software.

Raw sequence data (.bcl files) generated from Illumina HiSeq were converted into FASTQ files and demultiplexed using Illumina's bcl2fastq 2.17 software. One mismatch was allowed for index sequence identification.

For transcriptome downstream analysis, genes were processed with iDEP.96 (90, 91) that used EdgeR and average linkage and distance correlation to obtain hierarchical clustering of the top 1000 most variable genes after normalization of genes and samples. PGSEA within iDEP software was completed to obtain coherently altered pathways using Gene Ontology (92). The multifactor analysis plot shows log(fold changes) and average expression of genes with an FDR of less than 0.05 between the 2 conditions. Alternatively, a volcano plot is shown comparing $log_2$(fold changes) and *P* values with upregulated genes in red and downregulated genes in black. These were then analyzed by the ToppGene Functional Annotation tool (ToppFun) (93, 94) for enrichment in the top 50 substantial functional pathways that include databases from Biosystems (95); KEGG (96); Biosystems: Pathway Interaction Database; Biosytems: Reactome, MSigDB C2 Biocarta (v7.5.1) (97); and Panther DB (98).

*Single-cell RNA sequencing*. Single-cell RNA sequencing was performed from 10,000 single cells using standard 10× Genomics sample preparation of single nuclei for RNA sequencing, as described in the user manual. Briefly, a single-cell suspension was obtained from lungs of eosinophil-sufficient or -deficient BALB/c→C57BL/6$^{iPHIL}$ lung allografts (treated with DT or saline), as described above. Single nuclei isolation from tissue was performed based on established methodology.

Libraries were synthesized from lysed single nuclei. Within each droplet, RNAs were reverse transcribed into complementary DNA (cDNA) using the 10× Chromium Single Cell 5′ Library & Gel Bead Kit (v2) (10× Genomics) following the manufacturer's protocol. cDNA concentration was first measured by a Qubit fluorometer (Thermo Fisher Scientific) and 20 μL (approximately 20 ng total) was added to each reaction. After breaking the emulsion, cDNAs were amplified and fragmented, followed by the addition of Illumina adapters using the 10× Chromium Single Cell 5′ Library & Gel Bead Kit (v2) according to the manufacturer's instructions. Reactions were performed at 98°C for 45 seconds, 16 cycles at 98°C for 20 seconds, 67°C for 30 seconds, 72°C for 1 minute, followed by 72°C for 1 minute and then holding at 4°C. Following PCR, cDNA quantity was assessed by Qubit fluorometer. Bioanalyzer (Agilent) analysis and gel electrophoresis were used to determine the expected size after adapter ligation. The multiplexed libraries were pooled and sequenced on the S4 flow cell of NovaSeq 6000 (Illumina); 150-bp paired-end sequencing was performed.

For analysis, feature and barcode matrices of eosinophil-depleted and -sufficient mouse lung samples were imported into BBrowser 3.5.26 (BioTuring) based on established methodology (https://www.biorxiv.org/content/10.1101/2020.12.11.414136v1.full). After quality filtering, 17,265 cell profiles were obtained, including 8143 profiles from eosinophil-depleted and 9122 profiles from eosinophil-sufficient mice. Dimensionality reduction using t-distributed stochastic neighbor embedding (t-SNE) with canonical correlation analysis subspace alignment and unsupervised graph-based clustering was carried out. Analysis of representative marker genes identified clusters of specific pulmonary cell types in these samples. Data were exported from BBrowser into Seurat v4.3.0 (91) for visualization of features and comparative analyses of eosinophil-depleted and -sufficient samples.

*Flow cytometry*. All flow cytometric analysis was performed using saturating concentrations of fluorochrome-conjugated antibodies. All antibodies were purchased from BD Biosciences, BioLegend, or eBioscience (Thermo Fisher Scientific). Surface antibodies used were FITC anti–PD-1 (clone J43), FITC anti-CD62L (clone MEL-14), FITC anti-CD43 (clone eBioR2/60), FITC anti-CD38 (clone 90), FITC anti-CD4 (clone GK1.5), FITC anti-CD8a (clone 53-6.7), FITC anti–Gr-1 (clone RB6-8C5), FITC anti-F4/80 (clone BM8), PE anti-CD138 (clone 281-2), FITC anti-CD90 (clone 30-H12), PE anti–Siglec-F (clone 1RNM44N), PE anti-CD23 (clone B3B4), PerCP-Cy5.5 anti-CD45R (clone RA3-6B2), PerCP-Cy5.5 anti-GL7 (clone GL7), PerCP-Cy5.5 anti-IgM (clone RMM-1), PerCP-Cy5.5 anti-CXCR5 (clone L138D7), PerCP-Cy5.5 anti-CD44 (clone IM7), PE-Cy7 anti-IgD (clone 11-26c), APC anti-IgD (clone 11-26c.2a), APC anti-CCR3 (clone J073E5), Alexa Fluor 700 anti-CD5 (clone 53-7.3), APC-Cy7 anti-CD21/CD35 (clone 7-E9), APC-Cy7 anti-CD38 (clone 90), APC-e780 anti-CD11b (clone M1/70), BV421 anti–Siglec-F (clone E50-2440), eFluor 450 anti-CD45.2 (clone 104), BV421 anti-CD19 (clone 6D5), eFluor 450 anti-NK1.1 (clone PK136), BV605 anti-CD19 (clone 6D5), BV711 anti–γδ TCR (clone GL3), BUV496 anti-CD3 (clone 145-2C11), and BUV805 anti-CD4 (clone GK1.5).

Intracellular antibodies used were Alexa Fluor 488 anti–RORγt (clone B2D), PE anti-Ki67 (clone SolA15), PE anti-Foxp3 (clone FJK-16s), PE anti–IFN-γ (clone XMG1.2), PE anti-IRF4 (clone IRF4.3E4), PerCP-Cy5.5 anti–T-bet (clone eBio4B10), PE-Cy7 anti-GATA3 (clone TWAJ), APC anti–Blimp-1 (clone 5E7), APC anti-Foxp3 (clone FJK-16s), and APC anti-Bcl6 (clone BCL-DWN).

For determination of serum alloantibody titers, 200-μL aliquots of PBS with 0.5% BSA and 0.02% sodium azide (PBA) containing 2 × $10^6$ BALB/c or B6 thymocytes were mixed with 200 μL of serially diluted serum for 1 hour at 4°C. After 3 washes with PBA, cells were stained with polyclonal fluorochrome-conjugated goat anti–mouse IgM or anti–mouse IgG (Jackson ImmunoResearch) for 30 minutes at 4°C.

All flow cytometry experiments followed surface staining or intracellular staining protocols. For some experiments, mice were injected with 500 μg brefeldin 6 hours before tissue harvest; other sample collections were as described above. Samples were acquired on an Aurora (Cytek Biosciences) and analyzed using FlowJo v10.

*ELISPOT*. $CD4^+$ T cells (2 × $10^5$), purified from the lungs of CSB-treated BALB/c→C57BL/6 transplants depleted of eosinophils by anti-CCR3 treatment or not (IgG control), were cultured with irradiated stimulators (6 × $10^5$) (syngeneic H-2Kb, allogeneic H-2Kd) at a 1:3 ratio in plates coated with capture mouse IL-2–specific monoclonal antibody (Cellular Technology Limited). After 24 hours, cells were incubated with biotinylated IL-2–specific antibody and plates developed per manufacturer's instructions.

*ELISA*. Levels of mouse IFN-γ in lungs and draining lymph nodes were measured using ELISA kits (R&D Systems, DY485) per manufacturer's instructions.

*Statistics*. Data were analyzed with Student's *t* tests or 1-way ANOVA with post hoc tests for comparisons depending on Gaussian distribution and SDs, using multiple comparisons for comparison to control or between all groups. Data are presented as mean ± SEM. Differences were considered significant at *P* less than 0.05. Data visualization in all figures was accomplished by GraphPad Prism 8.1.

*Study approval*. All animal experiments were performed in accordance with protocols approved by the University of Maryland School of Medicine Institutional Animal Care and Use Committee (no. AUP-00000076).

*Data availability*. All underlying data can be accessed in the supplemental Supporting Data Values file. The bulk sequencing and single-cell RNA sequencing data have been deposited to the NCBI Gene Expression Omnibus database (GEO GSE223352 and GSE223268). The data that support the findings of this study are available from the corresponding author upon reasonable request.

## Author contributions

ASK and ZM conceived the study, designed experiments, interpreted the data, and wrote the manuscript. ZM, MAK, YG, and DL conducted most of the study. MAK performed flow cytometry experiments. YG and YY contributed to microsurgery experiments. DL and LL performed immunohistochemistry. AB, CK, and KC analyzed data. YT, IGL, and SPA performed ELISA, ELISPOT, and data analysis. JPC contributed to bioinformatic data analysis. CLL, SPA, AEG, DK, and EAJ contributed to manuscript revision and supplied constructive suggestions on interpreting results. All authors reviewed the final manuscript.

## Acknowledgments

ASK, AEG, and DK are supported by NIH grant P01 AI116501. ASK and EAJ are further supported by NIH grants R01 AI145108-01 and R01 HL166402. ASK is also supported by VA Health System grant I01 BX002299-05. IGL is supported by NIH grant R01 AR077562, AEG and DK are further supported by NIH grant R01 HL09601, and CLL is supported by NIH grant R01 HL128492. This work is partially supported by Chuck and Mary Meyers and Richard and Eibhlin Henggeler. ASK is also supported by the Lopker Family Foundation. The authors thank the services of the University of Maryland School of Medicine's and Greenebaum Comprehensive Cancer Center's Flow Cytometry Core, Baltimore, Maryland. This publication was supported by funds through the Maryland Department of Health's Cigarette Restitution Fund Program and the National Cancer Institute Cancer Center Support Grant (CCSG) P30CA134274. Illustrations provided by Anita Impagliazzo (anitaimedicalart@gmail.com) (www.anitaimedicalart.squarespace.com).

Address correspondence to: Alexander Sasha Krupnick, University of Maryland, 29 South Greene Street, Suite 504, Baltimore, Maryland 21201, USA. Phone: 410.328.6366; Email: AKrupnick@som.umaryland.edu.

1. Witt CA, et al. Lung transplant immunosuppression - time for a new approach? *Expert Rev Clin Immunol*. 2014;10(11):1419–1421.
2. Martinu T, et al. Acute cellular rejection and humoral sensitization in lung transplant recipients. *Semin Respir Crit Care Med*. 2010;31(2):179–188.
3. Lobo LJ, et al. Donor-specific antibodies are associated with antibody-mediated rejection, acute cellular rejection, bronchiolitis obliterans syndrome, and cystic fibrosis after lung transplantation. *J Heart Lung Transplant*. 2013;32(1):70–77.

4. Witt CA, et al. Acute antibody-mediated rejection after lung transplantation. *J Heart Lung Transplant*. 2013;32(10):1034–1040.
5. Djamali A, et al. Diagnosis and management of antibody-mediated rejection: current status and novel approaches. *Am J Transplant*. 2014;14(2):255–271.
6. Levine DJ, et al. Antibody-mediated rejection of the lung: a consensus report of the International Society for Heart and Lung Transplantation. *J Heart Lung Transplant*. 2016;35(4):397–406.
7. Sis B, Halloran PF. Endothelial transcripts uncover a previously unknown phenotype: C4d-negative antibody-mediated rejection. *Curr Opin Organ Transplant*. 2010;15(1):42–48.
8. Sarwal M, et al. Molecular heterogeneity in acute renal allograft rejection identified by DNA microarray profiling. *N Engl J Med*. 2003;349(2):125–138.
9. Berek C. Eosinophils can more than kill. *J Exp Med*. 2018;215(8):1967–1969.
10. Jacobsen EA, et al. Eosinophils: singularly destructive effector cells or purveyors of immunoregulation? *J Allergy Clin Immunol*. 2007;119(6):1313–1320.
11. Weller PF, Spencer LA. Functions of tissue-resident eosinophils. *Nat Rev Immunol*. 2017;17(12):746–760.
12. Le Moine A, et al. IL-5 mediates eosinophilic rejection of MHC class II-disparate skin allografts in mice. *J Immunol*. 1999;163(7):3778–3784.
13. Foster PF, et al. Blood and graft eosinophilia as predictors of rejection in human liver transplantation. *Transplantation*. 1989;47(1):72–74.
14. Braun MY, et al. IL-5 and eosinophils mediate the rejection of fully histoincompatible vascularized cardiac allografts: regulatory role of alloreactive CD8(+) T lymphocytes and IFN-gamma. *Eur J Immunol*. 2000;30(5):1290–1296.
15. De Groen PC, et al. The eosinophil as an effector cell of the immune response during hepatic allograft rejection. *Hepatology*. 1994;20(3):654–662.
16. Dollinger MM, et al. Peripheral eosinophil count both before and after liver transplantation predicts acute cellular rejection. *Liver Transpl Surg*. 1997;3(2):112–117.
17. Barnes EJ, et al. Applications and limitations of blood eosinophilia for the diagnosis of acute cellular rejection in liver transplantation. *Am J Transplant*. 2003;3(4):432–438.
18. Hongwei W, et al. Eosinophils in acute renal allograft rejection. *Transpl Immunol*. 1994;2(1):41–46.
19. Kormendi F, et al. The importance of eosinophil cells in kidney allograft rejection. *Transplantation*. 1988;45(3):537–539.
20. Onyema OO, et al. Eosinophils promote inducible NOS-mediated lung allograft acceptance. *JCI Insight*. 2017;2(24):e96455.
21. Onyema OO, et al. Eosinophils downregulate lung alloimmunity by decreasing TCR signal transduction. *JCI Insight*. 2019;4(11):e128241.
22. Larsen CP, et al. Long-term acceptance of skin and cardiac allografts after blocking CD40 and CD28 pathways. *Nature*. 1996;381(6581):434–438.
23. Okazaki M, et al. A mouse model of orthotopic vascularized aerated lung transplantation. *Am J Transplant*. 2007;7(6):1672–1679.
24. Li W, et al. Lung transplant acceptance is facilitated by early events in the graft and is associated with lymphoid neogenesis. *Mucosal Immunol*. 2012;5(5):544–554.
25. Li W, et al. Bronchus-associated lymphoid tissue-resident Foxp3$^+$ T lymphocytes prevent antibody-mediated lung rejection. *J Clin Invest*. 2019;129(2):556–568.
26. Jacobsen EA, et al. Eosinophil activities modulate the immune/inflammatory character of allergic respiratory responses in mice. *Allergy*. 2014;69(3):315–327.
27. Grimaldi JC, et al. Depletion of eosinophils in mice through the use of antibodies specific for C-C chemokine receptor 3 (CCR3). *J Leukoc Biol*. 1999;65(6):846–853.
28. Brynjolfsson SF, et al. Long-lived plasma cells in mice and men. *Front Immunol*. 2018;9:2673.
29. Nutt SL, et al. The generation of antibody-secreting plasma cells. *Nat Rev Immunol*. 2015;15(3):160–171.
30. Angelin-Duclos C, et al. Commitment of B lymphocytes to a plasma cell fate is associated with Blimp-1 expression in vivo. *J Immunol*. 2000;165(10):5462–5471.
31. Minnich M, et al. Multifunctional role of the transcription factor Blimp-1 in coordinating plasma cell differentiation. *Nat Immunol*. 2016;17(3):331–343.
32. Chu VT, et al. Eosinophils are required for the maintenance of plasma cells in the bone marrow. *Nat Immunol*. 2011;12(2):151–159.
33. Kinashi T, et al. Cloning of complementary DNA encoding T-cell replacing factor and identity with B-cell growth factor II. *Nature*. 1986;324(6092):70–73.
34. Prince L, et al. Eosinophils recruited during pulmonary vaccination regulate mucosal antibody production. *Am J Respir Cell Mol Biol*. 2023;68(2):186–200.
35. Victora GD, Nussenzweig MC. Germinal centers. *Annu Rev Immunol*. 2012;30:429–457.
36. Fischer MB, et al. Dependence of germinal center B cells on expression of CD21/CD35 for survival. *Science*. 1998;280(5363):582–585.
37. Crotty S. T follicular helper cell differentiation, function, and roles in disease. *Immunity*. 2014;41(4):529–542.
38. Krupnick AS, et al. Central memory CD8$^+$ T lymphocytes mediate lung allograft acceptance. *J Clin Invest*. 2014;124(3):1130–1143.
39. Ryg-Cornejo V, et al. Severe malaria infections impair germinal center responses by inhibiting T follicular helper cell differentiation. *Cell Rep*. 2016;14(1):68–81.
40. Liu F, Whitton JL. Cutting edge: re-evaluating the in vivo cytokine responses of CD8$^+$ T cells during primary and secondary viral infections. *J Immunol*. 2005;174(10):5936–5940.
41. Swaminathan AC, et al. Advances in human lung transplantation. *Annu Rev Med*. 2021;72:135–149.
42. Gelman AE, et al. Cutting edge: acute lung allograft rejection is independent of secondary lymphoid organs. *J Immunol*. 2009;182(7):3969–3973.
43. Lakkis FG, et al. Immunologic ‘ignorance’ of vascularized organ transplants in the absence of secondary lymphoid tissue. *Nat Med*. 2000;6(6):686–688.
44. Du X, et al. Oridonin prolongs the survival of mouse cardiac allografts by attenuating the NF-κB/NLRP3 pathway. *Front Immunol*. 2021;12:719574.

45. Higazi H, et al. Loss of Jak2 protects cardiac allografts from chronic rejection by attenuating Th1 response along with increased regulatory T cells. *Am J Transl Res*. 2019;11(2):624–640.
46. Obara H, et al. IFN-gamma, produced by NK cells that infiltrate liver allografts early after transplantation, links the innate and adaptive immune responses. *Am J Transplant*. 2005;5(9):2094–2103.
47. Li XC, et al. On histocompatibility barriers, Th1 to Th2 immune deviation, and the nature of the allograft responses. *J Immunol*. 1998;161(5):2241–2247.
48. Mariotti J, et al. Graft rejection as a Th1-type process amenable to regulation by donor Th2-type cells through an interleukin-4/STAT6 pathway. *Blood*. 2008;112(12):4765–4775.
49. Amarnath S, et al. Host-based Th2 cell therapy for prolongation of cardiac allograft viability. *PLoS One*. 2011;6(4):e18885.
50. Robb CT, et al. Key mechanisms governing resolution of lung inflammation. *Semin Immunopathol*. 2016;38(4):425–448.
51. Leitch AE, et al. Relevance of granulocyte apoptosis to resolution of inflammation at the respiratory mucosa. *Mucosal Immunol*. 2008;1(5):350–363.
52. Yamamoto S, et al. Cutting edge: pseudomonas aeruginosa abolishes established lung transplant tolerance by stimulating B7 expression on neutrophils. *J Immunol*. 2012;189(9):4221–4225.
53. Kulkarni HS, et al. Pseudomonas aeruginosa and acute rejection independently increase the risk of donor-specific antibodies after lung transplantation. *Am J Transplant*. 2020;20(4):1028–1038.
54. Frost AE, et al. Hyperacute rejection following lung transplantation. *Chest*. 1996;110(2):559–562.
55. Sureshbabu A, et al. Autoantibodies in lung transplantation. *Transpl Int*. 2020;33(1):41–49.
56. Bery AI, Hachem RR. Antibody-mediated rejection after lung transplantation. *Ann Transl Med*. 2020;8(6):411.
57. Fuquay R, et al. Renal ischemia-reperfusion injury amplifies the humoral immune response. *J Am Soc Nephrol*. 2013;24(7):1063–1072.
58. Yalcintas A, et al. Risk factors of antibody-mediated rejection and predictors of outcome in kidney transplant recipients. *Exp Clin Transplant*. 2020;18(suppl 1):29–31.
59. Miyairi S, et al. Natural killer cells: critical effectors during antibody-mediated rejection of solid organ allografts. *Transplantation*. 2021;105(2):284–290.
60. Mai HL, et al. Antibody-mediated allograft rejection is associated with an increase in peripheral differentiated $CD28^-CD8^+$ T cells - analyses of a cohort of 1032 kidney transplant recipients. *EBioMedicine*. 2022;83:104226.
61. Yagisawa T, et al. In the absence of natural killer cell activation donor-specific antibody mediates chronic, but not acute, kidney allograft rejection. *Kidney Int*. 2019;95(2):350–362.
62. Kohei N, et al. Natural killer cells play a critical role in mediating inflammation and graft failure during antibody-mediated rejection of kidney allografts. *Kidney Int*. 2016;89(6):1293–1306.
63. Yamane H, Paul WE. Cytokines of the γ(c) family control $CD4^+$ T cell differentiation and function. *Nat Immunol*. 2012;13(11):1037–1044.
64. Campbell DJ, et al. Chemokines in the systemic organization of immunity. *Immunol Rev*. 2003;195:58–71.
65. Breitfeld D, et al. Follicular B helper T cells express CXC chemokine receptor 5, localize to B cell follicles, and support immunoglobulin production. *J Exp Med*. 2000;192(11):1545–1552.
66. Schaerli P, et al. CXC chemokine receptor 5 expression defines follicular homing T cells with B cell helper function. *J Exp Med*. 2000;192(11):1553–1562.
67. Ueno H, et al. Pathophysiology of T follicular helper cells in humans and mice. *Nat Immunol*. 2015;16(2):142–152.
68. Kerfoot SM, et al. Germinal center B cell and T follicular helper cell development initiates in the interfollicular zone. *Immunity*. 2011;34(6):947–960.
69. Choi YS, et al. ICOS receptor instructs T follicular helper cell versus effector cell differentiation via induction of the transcriptional repressor Bcl6. *Immunity*. 2011;34(6):932–946.
70. Nurieva RI, et al. Bcl6 mediates the development of T follicular helper cells. *Science*. 2009;325(5943):1001–1005.
71. Ballesteros-Tato A, et al. Interleukin-2 inhibits germinal center formation by limiting T follicular helper cell differentiation. *Immunity*. 2012;36(5):847–856.
72. Cai G, et al. A regulatory role for IL-10 receptor signaling in development and B cell help of T follicular helper cells in mice. *J Immunol*. 2012;189(3):1294–1302.
73. McCarron MJ, Marie JC. TGF-β prevents T follicular helper cell accumulation and B cell autoreactivity. *J Clin Invest*. 2014;124(10):4375–4386.
74. Obeng-Adjei N, et al. Circulating Th1-cell-type Tfh cells that exhibit impaired B cell help are preferentially activated during acute malaria in children. *Cell Rep*. 2015;13(2):425–439.
75. Lee SK, et al. Interferon-γ excess leads to pathogenic accumulation of follicular helper T cells and germinal centers. *Immunity*. 2012;37(5):880–892.
76. Benckert J, et al. The majority of intestinal $IgA^+$ and $IgG^+$ plasmablasts in the human gut are antigen-specific. *J Clin Invest*. 2011;121(5):1946–1955.
77. Takemori T, et al. Generation of memory B cells inside and outside germinal centers. *Eur J Immunol*. 2014;44(5):1258–1264.
78. Smith KG, et al. The extent of affinity maturation differs between the memory and antibody-forming cell compartments in the primary immune response. *EMBO J*. 1997;16(11):2996–3006.
79. Jacobsen EA, et al. Eosinophil knockout humans: uncovering the role of eosinophils through eosinophil-directed biological therapies. *Annu Rev Immunol*. 2021;39:719–757.
80. Grisaru-Tal S, et al. Eosinophil-lymphocyte interactions in the tumor microenvironment and cancer immunotherapy. *Nat Immunol*. 2022;23(9):1309–1316.
81. Fallegger A, et al. TGF-β production by eosinophils drives the expansion of peripherally induced $neuropilin^-$ $ROR\gamma t^+$ regulatory T-cells during bacterial and allergen challenge. *Mucosal Immunol*. 2022;15(3):504–514.
82. Cosway EJ, et al. Eosinophils are an essential element of a type 2 immune axis that controls thymus regeneration. *Sci Immunol*. 2022;7(69):eabn3286.
83. Legrand F, et al. Human eosinophils exert TNF-α and granzyme A-mediated tumoricidal activity toward colon carcinoma cells. *J Immunol*. 2010;185(12):7443–7451.

84. Kanda A, et al. Eosinophil-derived IFN-gamma induces airway hyperresponsiveness and lung inflammation in the absence of lymphocytes. *J Allergy Clin Immunol*. 2009;124(3):573–582.
85. Fehniger TA, et al. Acquisition of murine NK cell cytotoxicity requires the translation of a pre-existing pool of granzyme B and perforin mRNAs. *Immunity*. 2007;26(6):798–811.
86. Spencer LA, et al. Human eosinophils constitutively express multiple Th1, Th2, and immunoregulatory cytokines that are secreted rapidly and differentially. *J Leukoc Biol*. 2009;85(1):117–123.
87. Davoine F, Lacy P. Eosinophil cytokines, chemokines, and growth factors: emerging roles in immunity. *Front Immunol*. 2014;5:570.
88. Dias FF, et al. Identification of piecemeal degranulation and vesicular transport of MBP-1 in liver-infiltrating mouse eosinophils during acute experimental Schistosoma mansoni infection. *Front Immunol*. 2018;9:3019.
89. Stewart S, et al. Revision of the 1996 working formulation for the standardization of nomenclature in the diagnosis of lung rejection. *J Heart Lung Transplant*. 2007;26(12):1229–1242.
90. Ge SX, et al. iDEP: an integrated web application for differential expression and pathway analysis of RNA-Seq data. *BMC Bioinformatics*. 2018;19(1):534.
91. Hao Y, et al. Integrated analysis of multimodal single-cell data. *Cell*. 2021;184(13):3573–3587.
92. Martens M, et al. WikiPathways: connecting communities. *Nucleic Acids Res*. 2021;49(d1):D613–D621.
93. Chen J, et al. ToppGene suite for gene list enrichment analysis and candidate gene prioritization. *Nucleic Acids Res*. 2009;37(web server issue):W305–W311.
94. Danopoulos S, et al. Transcriptional characterisation of human lung cells identifies novel mesenchymal lineage markers. *Eur Respir J*. 2020;55(1):1900746.
95. Geer LY, et al. The NCBI BioSystems database. *Nucleic Acids Res*. 2010;38(database issue):D492–D496.
96. Ogata H, et al. KEGG: Kyoto encyclopedia of genes and genomes. *Nucleic Acids Res*. 1999;27(1):29–34.
97. Liberzon A, et al. The Molecular Signatures Database (MSigDB) hallmark gene set collection. *Cell Syst*. 2015;1(6):417–425.
98. Mi H, et al. Protocol update for large-scale genome and gene function analysis with the PANTHER classification system (v.14.0). *Nat Protoc*. 2019;14(3):703–721.

## Version 1 02/08/2024

Electronic publication

